# An overview of the genus *Coprotus* (Pezizales, Ascomycota) with notes on the type species and description of *C. epithecioides* sp. nov.

**DOI:** 10.3897/mycokeys.29.22978

**Published:** 2018-01-12

**Authors:** Ivana Kušan, Neven Matočec, Margita Jadan, Zdenko Tkalčec, Armin Mešić

**Affiliations:** 1 Ruđer Bošković Institute, Bijenička cesta 54, HR-10000 Zagreb, Croatia

**Keywords:** *Coprotus epithecioides* sp. nov., *Coprotus sexdecimsporus*, Ascomycota, identification key, phylogeny, taxonomy

## Abstract

In a mycological research performed in the Sjeverni Velebit National Park, Croatia, a new species of *Coprotus* was discovered, described here as *C. epithecioides*. Along with the microscopic examination, phylogenetic analysis of the type material, based on ITS and LSU sequences, was performed in order to evaluate its relationship with the type species, *C. sexdecimsporus*. The type species was sequenced in this study for the first time, providing ITS and LSU sequences from two separate collections which displayed differences in macroscopic characters and content of paraphyses. An extended description of *C. sexdecimsporus* based on Croatian material is also provided. A worldwide identification key to the species assigned to the genus *Coprotus* is presented, along with a species overview, containing a data matrix. The phylogenetic position of *Coprotus* in the *Boubovia-Coprotus* clade within *Pyronemataceae* s.l. is discussed. *Coprotus sexdecimsporus* is also reported here as new to the Croatian mycobiota.

## Introduction

The name *Coprotus* Korf was first mentioned but not validly published by Korf (1954) as a seggregate of the genus *Ascophanus* Boud. (Boudier 1869) for species having iodine negative asci, hooked paraphyses and small guttulate spores. Kimbrough (1966) recognized a “*Coprotus* group” in *Ascophanus* Boud. with species that have iodine negative asci staining uniformly in Congo red and ascospores with de Bary bubbles. The genus *Coprotus* Korf & Kimbr. was validated by Kimbrough and Korf (1967), encompassing certain species of *Ascophanus* and *Ryparobius* Boud. (Boudier 1869), with *Coprotus sexdecimsporus* (P. Crouan & H. Crouan) Kimbr. & Korf chosen as the type species. Eckblad (1968) implied that *Leporina* Velen. (Velenovský 1947) should be the correct name instead of *Coprotus*, since the type specimen of *Leporina multispora* Velen. was found to be identical to *Ryparobius sexdecimsporus* (P. Crouan & H. Crouan) Sacc. This nomenclatural problem was elaborated by Kimbrough (1970), who concluded that the name *Leporina* should be rejected and *Coprotus* retained because the type material consists of mixed collections belonging to three different genera while the protologue contains “two or more entirely discordant elements”. The name *Coprotus* was put on a without-prejudice list of generic names of fungi for protection under the International Code of Nomenclature for algae, fungi and plants (Kirk et al. 2013).

Species of the genus *Coprotus* are characterised by oblate to lenticular or discoid, glabrous, translucent or whitish to yellow apothecia with coprophilous ecology. Asci are functionally operculate, non-amyloid, eight- to 256-spored, producing hyaline, smooth, eguttulate ascospores, containing gaseous inclusions referred to as de Bary bubbles when placed in anhydrous conditions. Paraphyses are filiform, mostly bent to uncinate and/or swollen at the apex, hyaline or containing pigment. The excipulum is composed primarily of globose to angular cells (Kimbrough et al. 1972).

The genus *Coprotus* was placed in the tribe Theleboleae (family Pezizaceae) by Kimbrough and Korf (1967). In later classifications Eckblad (1968) and Kimbrough et al. (1972) placed this genus into the family Thelebolaceae (Pezizales). Kish (1974) performed cytological and ontogenetical research on *C. lacteus* (Cooke & W. Phillips) Kimbr., Luck-Allen & Cain using axenic cultures, and concluded that this species shows much closer affinities with the Pyronemataceae *sensu* Eckblad (1968) than the Thelebolaceae. Study of the apical apparatus in *C. winteri* (Marchal & É.J. Marchal) Kimbr. and *C. lacteus* by Samuelson (1978) supported this view. Using transmission electron microscopy, Van Brummelen (1998) determined that the fine ascal structure of the wall and operculum in *C. lacteus* is characteristic of members of the Pyronemataceae s.l. Contrary to the mentioned micromorphological and cytological evidences, all members of the Thelebolaceae, including *Coprotus*, were placed in the class Leotiomycetes (Kirk et al. 2008, Lumbsch and Huhndorf 2010).

The phylogenetic affinity of *Coprotus* was studied using molecular data by Hansen et al. (2013), who showed that the genus belongs to the Pezizomycetes and forms a strongly supported monophyletic group with *Boubovia* Svrček (Pyronemataceae). This was confirmed by Lindemann et al. (2015) and Lindemann and Alvarado (2017). Wijayawardene et al. (2017) placed the genus *Coprotus* in the family Ascodesmidaceae (Pezizales, Pezizomycetes), and included 29 species. Additionally, isozyme analysis performed by Suárez et al. (2006) and RAPD patterns analysed by Ramos et al. (2008) detected a high intra-specific homogeneity in three *Coprotus* species (*C. lacteus*, *C. niveus* and *C. sexdecimsporus*). Furthermore, the AFLP fingerprinting technique applied to the same three *Coprotus* species (Ramos et al. 2015) exhibited the highest level of intra-specific variability in *C. sexdecimsporus*.

We began our own study of the genus *Coprotus* with an integrated taxonomical approach aimed at the type species, relying on vital taxonomic and phylogenetic methods. Previously only *C. ochraceus* (P. Crouan & H. Crouan) J. Moravec was included in phylogenetic analyses (Hansen et al. 2013, Lindemann et al. 2015, Lindemann and Alvarado 2017). Our inventory study of fungi in the Sjeverni Velebit National Park was aimed also on fimicolous fungi resulting with a collection of a *Coprotus* species found on a chamois dung, *Rupicapra rupicapra*, that appeared different from all other known species in the genus.

## Materials and methods

### *Ex situ* monitoring

The apothecia collected with the substrate were used for microscopic studies and DNA extraction. The remaining material (together with the original substrate) was kept in closed plastic boxes in a refridgerator under low temperature (4–8 °C) and out of doors (ca. 10–25 °C) in dark and in diffuse sunlight conditions. Over a two month period these were monitored observing a turnover of two to several generations.

### Microscopic studies

Observations of apothecia were made using a stereomicroscope under magnifications up to 80×. Microscopic characters based on living cells and tissues (^*^) were recorded using vital taxonomy methods (Baral 1992), while those based on dead cells and tissues (^†^) were obtained from fixed fresh material. All described microscopic elements were observed in tap water (H_2_O); cytochemical and histochemical data were obtained using the procedure described by Kušan et al. (2015). Microscopic features were observed with transmission light microscopes (bright field, phase contrast and dark field) under magnifications up to 1000×. Drawings were made free hand to scale, and microphotographs were mostly taken with a DSLR camera mounted on the microscope’s trinocular tube. Characters of apothecial construction and hymenial elements were based on a minimum of five ascomata. Spore measurements were based on samples of 50 fully mature, normally developed, and randomly selected ascospores (from living material ejected from asci). Measurements were taken directly using an ocular micrometer and from microphotographs using PIXIMÈTRE software ver. 5.9 (Henriot and Cheype 2017) to an accuracy of 0.1 µm. Spore wall layers were named following Heim (1962), except perispore is used rather than exospore following Harmaja (1974). Length, width and length/width ratio (“Q” value) are given as: min. – stat. mode – max. where “min.” = minimum (lowest measured value), “stat. mode” = statistical mode, “max.” = maximum (highest measured value). Length/width ratio (without mode value) was also introduced for asci. Dried material and accompanying data for all treated collections were deposited at the Croatian National Fungarium (CNF) in Zagreb.

A dichotomous key for identification of all putative species of *Coprotus* is presented. It was compiled from data derived from the literature and from our own observations. The key, except in one case, contains data for both living and dead materials. In this way the key is comprehensive. Species/character overview tables, containing supplementary data not used in the key, are presented as an aid for reliable identification (Tables 2–6). Ascus and ascospore measurements, originating from published sources, are enhanced by those obtained by measuring the original microphotographs and drawings. Ascus and ascospore “Q” values, taken from published references, were calculated from the original microphotographs and drawings.

Additional abbreviations:

KOH = potassium hydroxide; IKI = Lugol’s solution; CRB = Brilliant Cresyl Blue; CR = Congo Red; CB = Cotton Blue; AC = Acetocarmine; MLZ = Melzer’s reagent.

### DNA extraction, PCR amplification and DNA sequencing

Total genomic DNA was extracted from samples using DNeasy Plant kit (Qiagen Inc., USA). The LSU sequences were amplified using primers LR0R and LR7 (Vilgalys and Hester 1990). The primers ITS1-F (Gardes and Bruns 1993) and ITS4 (White et al. 1990) were used for amplification of the ITS regions (ITS1-5.8S-ITS2). All PCR amplifications consisted of 25-µL reaction volumes containing 0.2 mM of each dNTP, 0.2 µM of each primer, 1 U of Taq polymerase, 1.5 mM of MgCl and ~ 50 ng DNA. The touch-down PCR cycling profile consisted of initial 5 min at 95 °C, 10 cycles of 45 s at 95 °C, 45 s at 60 °C (decreasing 1 °C/cycle), 90 s at 72 °C, 25 cycles of 45 s at 95 °C, 45 s at 52 °C, 90 s at 72 °C, with final extension of 7 min at 72 °C. PCR products were sequenced in both directions using the same primers as for PCR by Macrogen (Macrogen Inc., Seoul, Korea). All sequences were deposited in GenBank (Table 1).

**Table 1.** Specimens used in this study with voucher information and GenBank accession numbers. Sequences produced by this study are indicated in bold.

Species	Voucher / strain number	ITS	LSU
*Aleuria aurantia*	OSC 100018	DQ491495	AY544654
*Anthracobia macrocystis*	OSC 100026	–	AY544660
*Ascobolus crenulatus*	KH.02.005(C)	DQ491504	AY544678
*Ascodesmis nigricans*	CBS 389.68	–	DQ168335
*Boubovia luteola*	R.K. 94/05	KX592793	KX592805
*Boubovia nicholsonii*	CNF 2/9121	**MG593545**	**MG593546**
*Boubovia ovalispora* (as *Pulvinula ovalispora* in NCBI)	BTO 95206 (C)	–	DQ220394
*Boubovia* sp.	M.H. 80813	KP309839	KP309876
*Byssonectria deformis*	N.V. 2009.04.09	KP309843	KP309866
*Coprotus epithecioides*	CNF 2/10450	**MG593539**	**MG593540**
*Coprotus ochraceus*	JHP-06.121 (C)	–	KC012673
*Coprotus sexdecimsporus* (1)	CNF 2/8942	**MG593541**	**MG593542**
*Coprotus sexdecimsporus* (2)	CNF 2/4928	**MG593543**	**MG593544**
*Cephaliophora irregularis*	ITS from YG-C22; LSU from CBS 218.62	KX683420	KC012668
*Cheilymenia stercorea*	OSC 100034	DQ491500	AY544661
*Eleutherascus lectardii*	CBS 626.71	–	DQ470966
*Geopora cooperi*	ITS from 16977; LSU from BAP 517 (FH)	JF908023	KC012678
*Geopyxis carbonaria*	PRM149720	KU932495	KU932547
*Geopyxis delectans*	KH.04.56a (FH)	KU932505	KU932555
*Glaziella aurantiaca*	PR 6376 (FH)	–	KC012681
*Heydenia alpina*	isolate 0732	HQ688653	HQ596526
*Humaria hemisphaerica*	ITS from KH.03.100 (FH); LSU from KH.03.10 (FH)	DQ200832	KC012683
*Hydnocystis piligera*	AH39303	JN048886	JN048881
*Lasiobolidium spirale*	CBS 782.70	–	FJ176873
*Lasiobolus ciliatus*	KS-94-005 (C)	–	DQ167411
*L. cunculi*	C F-54526 (C)	–	DQ168338
*Miladina lecithina*	KH.03.156 (FH)	–	DQ220371
*Paurocotylis pila*	Trappe 12583 (OSC)	KU932506	DQ168337
*Peziza vesiculosa*	TL-6398 (C)	AF491623	AF378367
*Pseudaleuria quinaultiana*	OSC 45766	EU669387	EU669429
*Pseudoboubovia benkertii*	N.V. 2006.12.04	KP309854	KP309874
*Pseudombrophila danuviana* (as *Kotlabaea danuviana* in NCBI)	isolate 6483 (B, Collection Benkert)	KX592794	KX592806
*Pseudombrophila theioleuca*	C F-70057 (C)	–	DQ062989
*Pulvinula constellatio*	N/A for ITS; KH.03.64 (FH) for LSU	AF289074	DQ062987
*Pulvinula convexella*	KH.01.020 (C)	–	DQ062986
*Pulvinula niveoalba*	M.A.R. 290809 27	KX592796	KX592808
*Pyronema domesticum*	OSC 100503 (strain CBS 666.88)	DQ491517	DQ247805
*Scutellinia scutellata*	OSC 100015	DQ491492	DQ247806
*Sowerbyella imperialis*	KH.09.222	KJ619953	KJ619950
*Stephensia bombycina*	Trappe 3268 (OSC, FH)	KU932484	DQ220435
*Tarzetta catinus*	KS-94-10A (C)	DQ200833	DQ062984

### Phylogenetic analyses

A data matrix for alignment was constructed. Phylogenetic analyses included eight newly identified sequences from this study, along with the sequences retrieved from GenBank (Table 1), *viz.*: Amicucci et al. (2001), Hansen et al. (2001), Hansen et al. (2002), Hansen et al. (2005), James et al. (2006), Schoch et al. (2006), Spatafora et al. (2006), Tedersoo et al. (2006), Perry et al. (2007), Schoch et al. (2009), Alvarado et al. (2011), Leuchtmann and Clémençon (2012), Hansen et al. (2013), Osmundson et al. (2013), Lindemann et al. (2015), Ghosta et al. (2016), Wang et al. (2016), Lindemann and Alvarado (2017). Newly sequenced material included one *Coprotus epithecioides* collection, two *C. sexdecimsporus* collections and one *Boubovia nicholsonii* collection (FRANCE. Nouvelle-Aquitaine, Charente-Maritime, Saint Savinien, 23 km E-SE from Rochefort, 10 m a.s.l.; on remnants and rotten branches and twigs with leaves of *Cupressus macrocarpa* lying on the heap, 22 Jan 2012, M. Hairaud and P. Tanchaud (CNF 2/9121, duplex M.H. 80112)). Sequences alignments were obtained using CLUSTAL W in BIOEDIT 7.0.5.3 (Hall 1999). A concatenated alignment of ITS + LSU was generated. The final alignment contained 1590 bp. The maximum likelihood analyses were performed using MEGA 6 (Tamura et al. 2013) with GTR + G + I model and 1000 bootstrap replicates to assess branch support. *Ascobolus crenulatus* was used as the outgroup. Besides the combined (ITS + LSU) analyses, the LSU dataset, with additional species (Table 1), was also generated. The LSU alignment consisted of 894 characters. The evolutionary history was inferred by using the maximum likelihood method based on the general time reversible model, with discrete gamma distribution and some sites evolutionary invariable (GTR + G + I). *Peziza vesiculosa* and *Ascobolus crenulatus* were used as outgroups. Branch support was assessed using 1000 bootstrap replicates. All analyses were performed in MEGA 6 software ver. 6.0 (Tamura et al. 2013).

## Results

### Phylogenetic analyses

**Figure 1.**
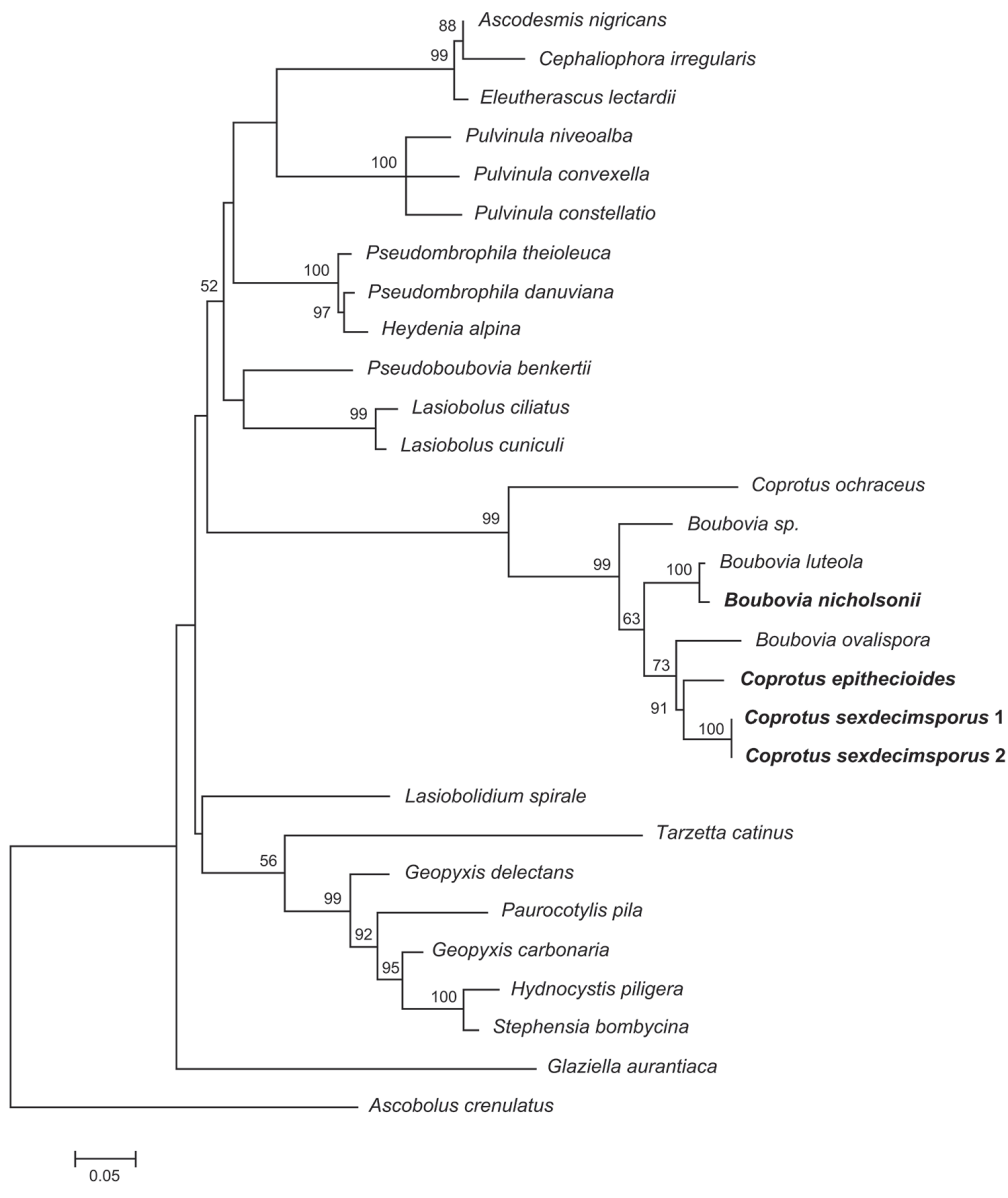
Maximum likelihood phylogenetic tree based on a concatenated ITS and LSU dataset. Sequences recovered during this study are shown in bold type. Bootstrap values greater than 50% are indicated at the nodes. *Ascobolus crenulatus* was used as the outgroup. The bar length indicates the number of nucleotide substitutions per site.

**Figure 2.**
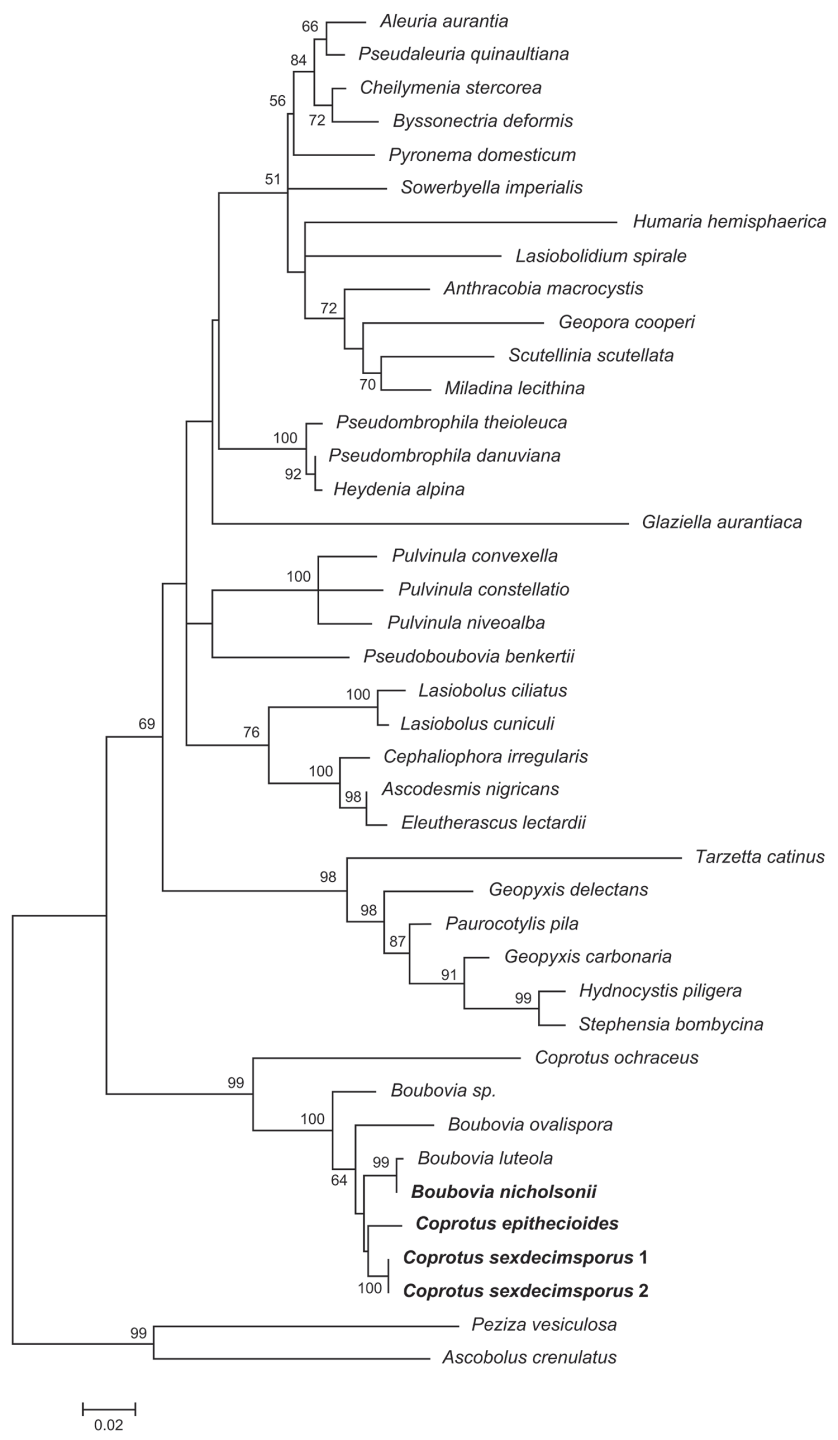
Maximum likelihood phylogenetic tree inferred from the LSU dataset. Sequences recovered during this study are shown in bold type. Bootstrap values greater than 50% are indicated at the nodes. The tree was rooted to *Peziza vesiculosa* and *Ascobolus crenulatus*. The bar length indicates the number of nucleotide substitutions per site.

The ITS + LSU alignment consisted of 1590 characters including gaps, of which 763 were conserved, 777 were variable, and 230 were parsimony informative. The LSU alignment consisted of 894 characters including gaps, of which 32 were conserved, 319 were variable, and 224 were parsimony informative. The type species *Coprotus sexdecimsporus* was sequenced for the first time to ascertain the real phylogenetic position of the genus *Coprotus*. The two phylogenies (based on LSU, and concatenate analysis of LSU and ITS) firmly nested the *Coprotus* species in the order Pezizales, as a member of the *Boubovia-Coprotus* lineage inside the Pyronemataceae s.l., in a species group next to the *Geopyxis-Tarzetta* and *Ascodesmis-Pulvinula* clades (but without high support in our contracted analyses, Figs 1, 2). In both phylogenetic trees, species in the genera *Boubovia* and *Coprotus* were clustered together, with high support values. *Coprotus ochraceus* showed a distant relationship to the type species *C. sexdecimsporus* as a phylogenetically earlier diverging lineage. Our newly described species appeared closely related to the type species. The two collections of *C. sexdecimsporus* sequenced displayed 100% sequence identity (ITS and LSU).

## Taxonomy

### Coprotus

Taxon classificationFungiThelebolalesThelebolaceae

Korf & Kimbr., American Journal of Botany 54(1): 21, 1967.

[≡ Coprotus Korf, Rapports et communications VIII Congrès International de Botanique I 1954 (sect. 18/20): 80, 1954, *nomen nudum*]

#### Type species.

*Coprotus sexdecimsporus* (P. Crouan & H. Crouan) Kimbr. & Korf.

As presently circumscribed, the genus *Coprotus* is clearly characterised by the following combination of characters: obligate coprophilous ecology, eugymnohymenial apothecial development, apothecia with reduced marginal tissue without setose hairs; inamyloid asci uniformly stainable in CR, with functional operculum; prolate, smooth (under transmission light microscope), eguttulate ascospores in all developmental stages sporoplasm of which have strong affinities to form de Bary bubble in any anhydrous conditions (especially in media such Cotton Blue). Mature spores ejected from living asci possess temporary thick and gelatinous sheath. Anamorph not known.

### Coprotus sexdecimsporus

Taxon classificationFungiThelebolalesThelebolaceae

(P. Crouan & H. Crouan) Kimbr. & Korf, American Journal of Botany 54(1): 22, 1967.

Fig. 3

≡ Ascobolus sexdecimsporus P. Crouan & H. Crouan, Annales des Sciences Naturelles Botanique ser. 4., 10: 195, 1858.
≡ Ascophanus sexdecimsporus (P. Crouan & H. Crouan) Boud., Annales des Sciences Naturelles Botanique ser. 5., 10: 247, 1869.
≡ Ryparobius sexdecimsporus (P. Crouan & H. Crouan) Sacc., Sylloge Fungorum 8: 541, 1889.

#### Description.

*Apothecia* not confluent, circular from the top view, at first globular, then flattened-turbinate and finally lenticular from the side view, sessile, evenly hyaline to creamy white or translucent pale greyish-rosy (if subjected to strong insolation), glabrous, ^*^0.1–0.5 mm in diameter, solitary to gregarious. Hymenium granulose due to the protrusion of living mature asci, concolorous with excipular surface, matte. Margin rounded in vertical median section, entire, smooth, not raised above hymenial plane. Outer surface smooth, concolorous with the hymenium. Subicular hyphae indistinguishable. *Hymenium* ^*^95–140 µm thick. *Asci* clavate with truncate apex, ^*^84–143 × 21.4–29.6 µm, ^†^89–104 × 16.4–23.3 µm, ^*^Q = 4.1–5.6, significantly shorter and more clavate at the marginal rim, when mature ^*^protruding above hymenium up to 26 µm, *pars sporifera* ^*^47.3–63.3 µm, 16-spored, hyaline, base attenuated, bifurcate, arising from perforated croziers, only fully mature asci with flat lentiform operculum clearly delimited prior the spore discharge, ^*^6.6–8 µm in diam. and ^*^0.6 µm thick, lateral wall 3-layered, ^*^0.7–0.8 µm thick, after spore discharge operculum as a rule clearly visible; in IKI inamyloid; in CR outermost wall vividly rutile-red throughout the ascal length, median layer pale rutile-yellow, innermost layer greyish; in CB cyanophobic. *Ascospores* ^*^10.7–*11.7*–13.8 × 6.8–*7.9*–8.5 µm, ^*^Q = 1.4–*1.7*–1.7, ellipsoid to narrowly ellipsoid and most often radially symmetrical, with rounded-obtuse poles, rarely slightly bilaterally symmetrical with one side somewhat less convex but never flattened, 1-celled, hyaline; in living asci bi- to triseriate; when freshly ejected remain in a single group for a while due to the delicate sticky sheath enveloping individual spores; surface smooth; wall 3-layered, 0.6–0.7 µm thick, perispore dull, epispore brightly refractive, endospore layer with pale greyish-isabelline refractivity; in IKI no notable differential stainings; eguttulate, uninucleate, nucleus ±centrally to unipolarly positioned, 2.7–3 µm wide, in CRB nucleus and sheath more contrasted, perispore dull deep bluish-violet/deep cyan, epispore CRB-, endospore purplish lilac/medium violet; after applying KOH spore sheath dissolves instantly, all structures discoloured, perispore not loosening, endospore layer purplish-rosaceous; in CR perispore dull, not stained as epispore, but endospore lilac reddish; in AC completely devoid of staining; in CB de Bary bubbles present only in mature spores, perispore not loosening, weakly cyanophilic. *Paraphyses* cylindric, apically obtuse to subclavate, always slighty bent to uncinate, densely septate, rarely simple but often richly branched in the upper part; apically producing abundant medium to strongly refractive golden-yellow to cinnamon-yellow granular exudate, in IKI copper orange, in CRB dark grey blue, after applying KOH rubis red-grey; apical cells ^*^6.9–16.4 × 2–3.4 µm, ^†^1.4–2.8 µm wide, wall thin and hyaline, cells in the upper half contain minute medium to strongly refractive hyaline globules ^*^0.2–1 µm wide or in pigmented apothecia with beer-yellow to beer-orange scattered dotted granules which are in IKI greyish green, in CRB deep purplish-lilac to deep violet; in CB wall cyanophobic, cytoplasm weakly cyanophilic. *Subhymenium* only slightly differentiated from medullary excipulum, ^*^12–19 µm thick, composed of hyaline *textura globulosa-angularis*, cells ^*^3.8–7.5 µm wide. *Medullary excipulum* hyaline, in the middle flank ^*^12–22 µm thick, composed of *textura porrecta*, cells runing parallel to the surface, ^*^1.4–4.8 µm wide. *Margin* subhyaline, fairly reduced to a thin cellular zone ^*^9.6–11.3 µm thick at ½ of hymenium height, composed of small celled *textura angularis* 1–2 cell thick, cells clavate or elongated angular, 2.4–8.8 µm wide, marginal rim composed of prismatic terminal cells which do not protrude above hymenium; in CB cell walls strongly cyanophilic. *Ectal excipulum* hyaline, in the middle flank ^*^48–56 µm thick, composed of *textura globulosa*, cells ^*^7.2–16 µm wide, walls yellowish; in IKI some cells with visible moderate accumulations of glycogene; in CB cell walls slightly cyanophilic; in AC cell walls and cytoplasm deeply lilac. Overall excipulum devoid of crystalline matter, without colouring in KOH, in IKI completely inamyloid. Anamorph not found.

**Figure 3.**
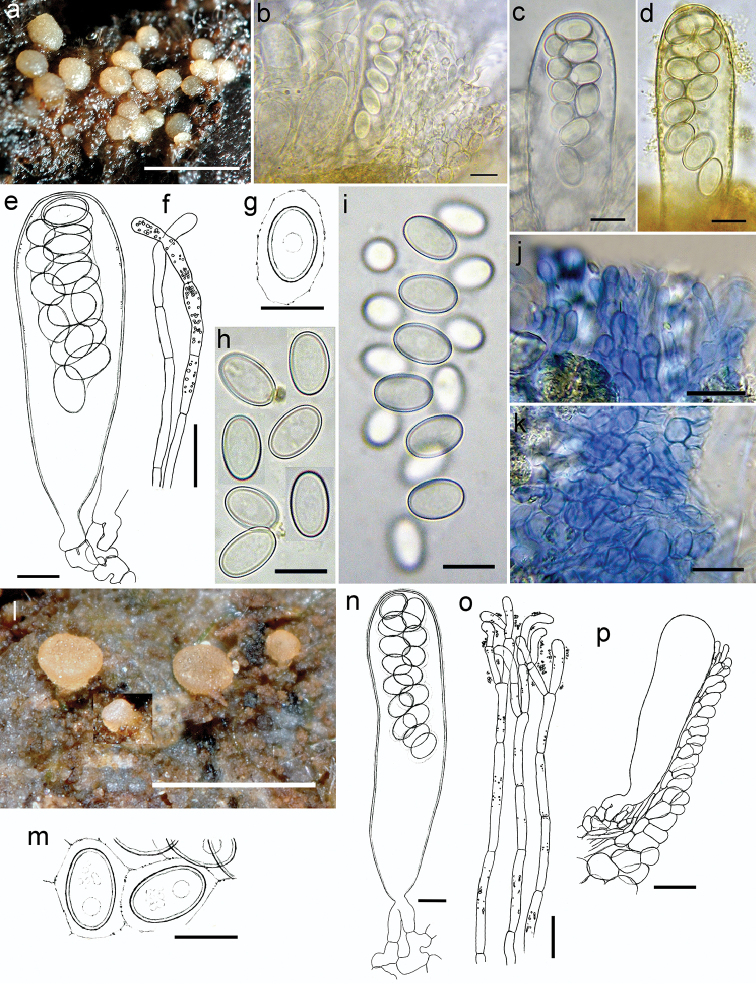
*Coprotus sexdecimsporus*. **a** Fresh apothecia on *Equus asinus* dung **b** Cross section with immature asci, paraphyses and marginal cells **c, d** Asci protruding above hymenium **e** Ascus with ascogenous cells **f** Paraphyses **g** Freshly ejected ascospore with a sheath **h** Mature ascospores **i** 16-spored freshly ejected packet of ascospores **j** Marginal cells from side view **k** Ectal excipulum cells in top view **l** Fresh apothecia on *Lepus europaeus* dung **m** Freshly ejected ascospores held together with a sheath **n** Ascus with ascogenous cells **o** Paraphyses with granular pigment and copious exudate **p** Excipular and marginal tissue. **b, c, e–g, i, m-p** ^*^tap water **d, h** ^*^IKI **j, k** ^†^CB **a–i** from CNF 2/8394 **j–p** from CNF 2/8942. Scale bars: **a, l** 1 mm, **b–k, m–o** 10 μm, **p** 20 μm; del. N. Matočec, phot. N. Matočec & I. Kušan.

#### Distribution and ecology.

The species has a cosmopolitan distribution and can be found on dung of various wild and domestic animals, mainly herbivores (especially ruminant animals and rodents). In the temperate zone it is distributed in the habitats from maritime to alpine zones.

#### Specimens examined.

CROATIA. Zadar County, Island of Dugi Otok, Velo jezero area, 5 km W from Sali, 43°56.46'N; 15°06.00'E, 5 m a.s.l., on dung of *Equus asinus*, 1 Jun 1998, N. Matočec (CNF 2/3806); Split-Dalmatia County, Island of Vela Palagruža, 70 m E-NE from the lighthouse, 42°23.58'N; 16°15.38'E, 60 m a.s.l., on dung of *Equus asinus*, 29 Mar 1999, N. Matočec (CNF 2/4200); Dubrovnik-Neretva County, Koprendol area, 7.5 km N-NE from Metković, 42°59.30'N; 17°37.44'E, 130 m a.s.l., on dung of *Ovis aries*, 5 Mar 2001, N. Matočec (CNF 2/4928); Dubrovnik-Neretva County, Peninsula Prevlaka (Oštra), 4.8 km N-NW from Vitaljina, 42°24.22'N; 18°30.53'E, 25 m a.s.l., on dung of *Equus asinus*, 31 Dec 2009, I. Kušan and N. Matočec (CNF 2/8394); Lika-Senj County, Sjeverni Velebit National Park, northern part of the Mt. Velebit, 280 m SW from the Vučjak peak (1644 m), 44°48.83'N; 14°58.46'E, 1550 m a.s.l.; on dung of *Lepus europaeus*, 11 Jun 2011, N. Matočec and I. Kušan (CNF 2/8942).

#### Notes.

De Sloover (2002) summarises the data on the distribution of pigments in microscopic elements in the *Coprotus* species described up to that time. His overview suggests that paraphyses are not the only cause of the overall apothecial pigmentation. However, our detailed study on living material of *C. sexdecimsporus* over a period of two months clearly showed that cytoplasmic pigments in the paraphyses develop with exposure to light. These observations used apothecia on original substrate and were carried out under controlled conditions. The pigments developed under sunlight or artificial light with a sufficient amount of the ultraviolet wave-length. On the other hand, pigmentation was completely absent if apothecia were grown continually under dark or low-light conditions. There is considerable variability in ascospore dimensions given in the literature. Although it seems that ascospore length may vary regardless of any presently visible cause, the ascospore diameter seems to be smaller in material from the Southern Europe / Mediterranean region. Accordingly, material from Italy (Doveri 2004) and Tunisia (Häffner 1996), almost completely overlap with our studied material from the East Adriatic region. These are in the range of ascospore widths from 6.9–8.5 μm. Specimens from the European Atlantic (Crouan’s material restudied by Le Gal, 1960), Norway (Aas, 1983) and both Americas (Kimbrough et al. 1972, Dokmetzian et al. 2005) have spores with greater spore widths, ranging from 7.5–10 μm. These differences might point to some ecological-geographical causes. The type material is missing according to Kimbrough et al. (1972).

### Coprotus epithecioides

Taxon classificationFungiThelebolalesThelebolaceae

Matočec & I. Kušan sp. nov.

823596

Figs 4 , 5

#### Type.

CROATIA. Lika-Senj County, Sjeverni Velebit National Park, northern part of the Mt. Velebit, Hajdučki kukovi area, 150 m W from Golubić peak (1650 m), 44°46.05'N; 15°00.88'E, 1580 m a.s.l.; on dung of chamois (*Rupicapra rupicapra*), 11 Oct 2017, I. Kušan (holotype CNF 2/10450, GenBank sequences ITS MG593539, LSU MG593540).

#### Etymology.

The specific epithet refers to epithecium-like ascal protective formation composed of swollen apical paraphyses cells.

#### Description.

*Apothecia* not confluent regularly circular to irregular from the top view, at first oblate, then turbinate, finally pulvinate from the side view, sessile, subhyaline to creamy grey or pale yellowish, glabrous, ^*^170–420 µm in diameter, solitary or gregarious. Hymenium only very finely scurfy, ascal protrusions not clearly visible. Margin rounded in vertical median section, entire and smooth, expanded with downwards positioned rim, never raised above hymenial plane. Outer surface smooth, concolorous with the hymenium. Subicular hyphae indistinguishable. *Hymenium* ^*^75–98 µm thick. *Asci* shortly cylindric with slightly truncate apex, ^*^60–74.8 × 13.4–15.5 µm, ^†^51.5–62 × 11.8–14 µm (Q = 3.8–5.2), when mature ^*^protruding above hymenium up to 7.5 µm, *pars sporifera* ^*^28–34 µm, 8-spored, hyaline; base attenuated, bifurcate, arising from perforated crosiers; only optimally oriented fully mature asci with flat lentiform operculum clearly delimited prior the spore discharge, ^*^6.3–6.6 µm in diam. and ^*^0.5 µm thick, lateral wall 3-layered, ^*^0.6 µm thick, after spore discharge operculum as a rule clearly visible; in IKI inamyloid; in CR outermost wall vividly rutile-red throughout the ascal length, median layer pale rutile-yellow, innermost layer greyish; in CB asci cyanophobic. *Ascospores* ^*^7.9–*8.8*–9.6 × 4.8–*5.2*–5.6 µm, ^†^8–*9.1*–9.5 × 4.2–*5*–5.2 µm, ^*^Q = 1.5–*1.6*–1.9, ^†^Q = 1.6–*1.9*–2.0, bilaterally symmetrical with one side flattened, subphaseoliform to phaseoliform, poles rounded, 1-celled; uni- to biseriate in living asci, freshly ejected remain in a group for a while due to the delicate subglobose sticky sheath enveloping individual spores; hyaline, smooth; wall 3-layered, 0.4 µm thick, perispore dull, epispore brightly refractive, endospore subhyaline, barely optically differentiated; eguttulate, uninucleate, nucleus always ±polarly positioned, 2.2–2.5 µm wide; in IKI perispore and epispore not stained, endospore purplish, nucleus slightly contrasted; in CRB without differential stainings, the edges of spore sheath sharply contrasted, after applying KOH spore sheath instantly dissolves, perispore not loosening, endospore layer purplish-rosaceous; in CB with one eccentrically positioned de Bary bubble in mature spores, perispore not loosening, moderately cyanophilic. *Paraphyses* ±densely septate, with thin, hyaline walls, cylindric in the lower part, often branched in the upper part, rarely simple, apically ±bent clavate or capitate, not producing copious exudate; of two types: (a) epithecioid, reaching higher level, with apical short and capitate cell, ^*^6.8–10 × 5–9.9 µm, ^†^6.2–11.2 × 4–8 µm, with 1–2 subapical cells often also swollen (moniliform), forming ±continuous layer above living immature asci, and (b) of usual type with elongated clavate apical cells, ^*^8.2–14.8 × 2.3–4.4 µm, ^†^5.5–11 × 2–3.3 µm; both types may contain yellow-orange pigment, often of crystalloid, fibrillar structure; pigment in IKI cinnamon-grey, in CRB purplish-lilac, often barely visible since mainly included in large globose, deeply stained blue-violet vacuole; in CB wall cyanophobic, cytoplasm pale greyish-blue. *Margin* reduced, composed of *textura globulosa-angularis*, cells not elongated, ^*^3.8–6 µm wide, cylindric-elongated cells absent; weakly cyanophilic in CB. *Subhymenium* hyaline, not differentiated from medullary excipulum. *Medullary excipulum* hyaline, in the central part ^*^32–56 µm thick, in the middle flank ^*^10–14 µm thick, composed of *textura epidermoidea*, cells thin-walled, ^*^2.3–4.8 µm wide, in CB cyanophobic. *Ectal excipulum* hyaline, in the middle flank ^*^17–22 µm thick, composed of *textura globulosa-angularis*, cells ^*^9.8–16.5 × 7.8–14.7 µm, ^†^4.5–12 × 2.3–9.5 µm, walls thickened, refractive, yellowish, ^*^0.5–0.7 µm thick, in CB cell walls slightly cyanophilic. Overall excipulum without crystalline matter, dextrinoid reaction in MLZ and colouring in KOH; in IKI inamyloid and devoid of glycogene accumulations. Anamorph not found.

**Figure 4.**
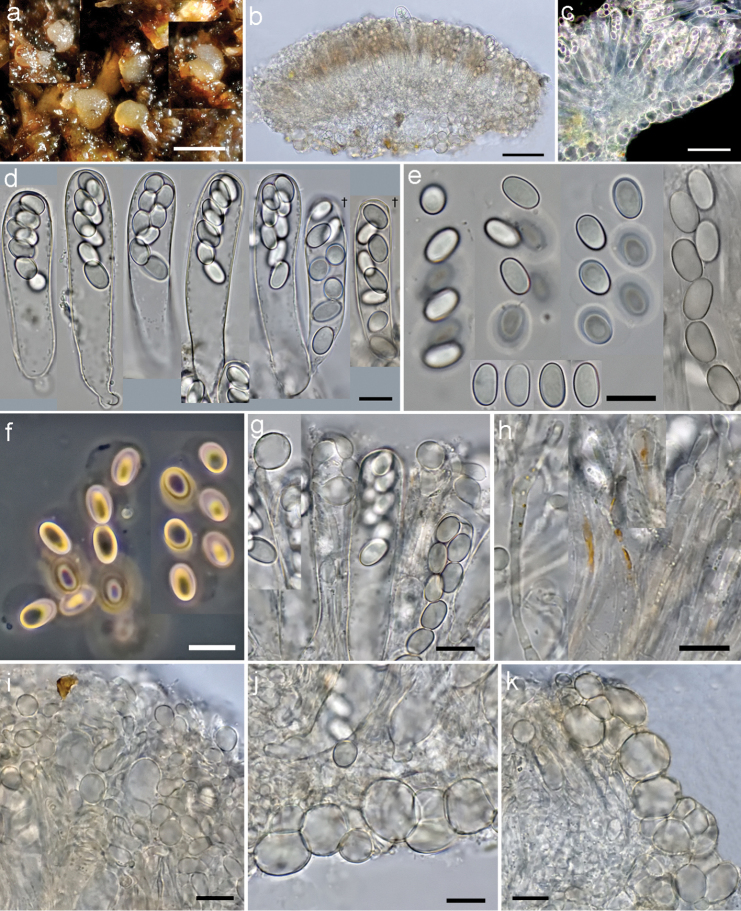
*Coprotus epithecioides* (CNF 2/10450, holotype). **a** Fresh apothecia on *Rupicapra rupicapra* dung **b** Cross section through the whole apothecia **c** Cross section in dark field **d** Asci **e** Freshly ejected ascospores glued together with a sheath and individual ascospores **f** Freshly ejected ascospores in phase contrast **g** Epithecioid paraphyses **h** Clavate paraphyses with pigment content **i** Epithecioid hymenial cover **j** Excipular flank **k** Marginal tissue. All elements observed in tap water and in living state, except two asci on **d** marked with a cross (^†^); Scale bars: **a** 0.5 mm, **b, c** 50 μm, **d–k** 10 μm, phot. N. Matočec & I. Kušan.

**Figure 5.**
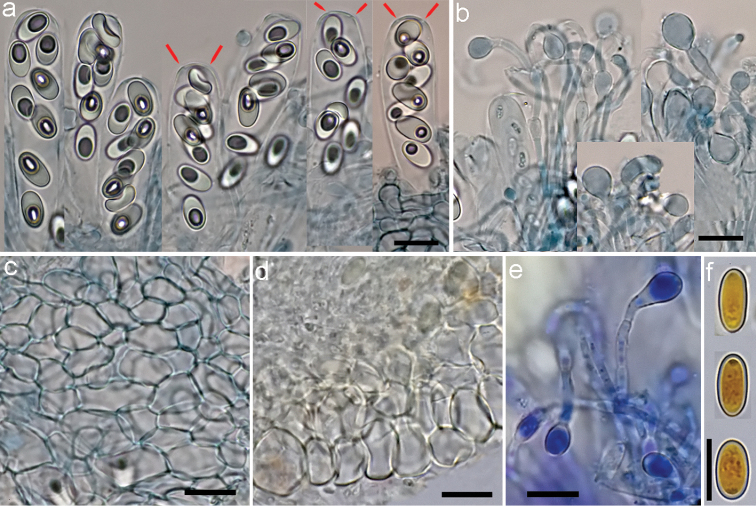
*Coprotus epithecioides* (CNF 2/10450, holotype). **a** Asci with ascospores containing de Bary bubbles, red markings show opercular delimitation **b** Paraphyses **c** Ectal excipulum from top view **d** Excipular flank **f** Paraphyses **g** Ascospores. **a–c** ^†^CB **d** ^†^MLZ **e** ^*^CRB **f** ^†^IKI. Scale bars: **a–f** 10 μm, phot. N. Matočec & I. Kušan.

#### Distribution and ecology.

The species is known so far only from Mt. Velebit, Croatia. The only collection originates from chamois dung in the alpine karstic habitat.

#### Other specimens examined.

None.

#### Notes.

*Coprotus epithecioides* has several characters making it distinct from other species in the genus. The paraphyses are of two types, along with the usual filiform-clavate ones, there are also an abundance of those with very short, swollen apical cells, that mutually form an epithecioid protective layer over immature asci, a character not recorded so far in the genus *Coprotus*. Additionally, in the epithecioid type, 1–2 subapical cells are often also swollen. This gives the paraphyses a moniliform appearance. When present, paraphysal pigments are most often orange to reddish-orange and crystalloid, i.e. of fibrillar shape, resembling the carotenoid pigmentation of *Scutellinia* species. Spores are highly bilaterally symmetric compared to *C. glaucellus*, *C. subcylindrosporus*, *C. argenteus* and *C. sexdecimsporus* (which has only inconspicuously and partly bilaterally symmetric spores) and the spores are significantly shorter than those of *C. subcylindrosporus*, *C. argenteus* and *C. sexdecimsporus*. *Coprotus glaucellus* differs by the presence of only apically uninflated to subclavate paraphyses which do not form an epithecioid protective cover over immature asci. Also it has notably elongated cells at the marginal edge. As elaborated above, paraphysal cytoplasmic pigments normally also develop in this species if the fungus is strongly exposed to sunlight or artificial light with ultraviolet wave-lengths. The pigmentation is completely absent if the apothecia is grown continually under dark or low-light conditions (see notes under *C. sexdecimsporus*).

##### Worldwide identification key to the putative species of the genus *Coprotus*

**Table:** 

1	Apothecial margin and/or upper flank beset with very long, paraphysis-like terminal cells, over 60 μm long, raising above hymenial plane	**2**
–	Apothecial margin not raised above hymenial plane, composed of ±isodiametric or somewhat elongated cells up to 25 μm long	**4**
2	Apothecial margin composed of large globose cells accompanied by greatly elongated cylindric-obtuse terminal cells on upper flank, up to 200 μm long; asci narrowly cylindric (Q ~10–11), 150–185 μm long; ascospores ellipsoid (Q = 1.5–1.9), 12.5–15.5 μm long; paraphyses broad cylindric, 6–9 μ wide	***C. arduennensis* J.R. De Sloover**
–	Apothecial margin devoid of globose cells, beset only with apically widened elongated terminal cells resembling paraphyses; asci cylindric to cylindric-ventricose (Q = 8.4–9.8), 70–100 μm long; ascospores narrowly to elongated ellipsoid (Q = 1.8–2.2), not exceeding 13.5 μm in length; paraphyses filiform, below 4 μ wide	**3**
3	Terminal cells on margin greater than 100 μm long; ascospores elongated ellipsoid (Q = 2.0–2.2), 8.5–10 × 4–5 μm; apothecia comparatively large, over 1 mm diam.	***C. marginatus* Kimbr., Luck-Allen & Cain**
–	Terminal cells on margin 60–95 μm long; ascospores narrowly ellipsoid (Q = 1.8–2), 10–13.5 × 6–7 μm; apothecia 290–650 μm diam.	***C. dhofarensis* Gené, El Shafie & Guarro**
4	Apothecia discoid or saucer shaped with complex excipular structure: medullary excipulum thick and sharply differentiated from the ectal layer, composed of *textura intricata*, ectal excipulum of *textura globulosa-angularis*; asci narrowly cylindric (Q > 10)	**5**
–	Apothecia principally subglobose, turbinate to pulvinate with excipular layers weakly or not differentiated, composed mostly of *textura globulosa-angularis*, with inner and marginal cells of gradually smaller diameter; asci stout (Q < 10)	**6**
5	Ectal excipular layer covered with cortical layer of elongated cylindric cells; asci 60–90 × 6–9 μm (Q = 10–11.5); ascospores elongated ellipsoid, 7–8.5 × 3.5–4.5 μm; paraphyses filiform, apically bent	***C. baeosporus* Jeng & J.C. Krug**
–	Ectal excipular layer composed only of large-celled *textura globulosa-angularis*; asci 163–200 × 10–16 μm (Q ~14); ascospores narrowly ellipsoid, 13.7–18 × 7.5–9 μm; paraphyses apically clavate, straight	***C. ochraceus* ss. Thind et al. (Thind et al. 1978)**
6	Apothecial margin composed of *texura globulosa-angularis* as in the excipular flanks, though cells gradually smaller	**7**
–	Apothecial margin composed of elongated, prismatic cells, 6–25 × 2–10 μm, and excipular flanks of *textura globulosa-angularis*	**11**
7	Asci cylindric (Q = 8.2–9.7), 85–150 × 9.0–17.5 μm; paraphyses filiform, 1.5–3 μm wide; apothecia markedly constricted below to a ±substipitate base	**8**
–	Asci broad clavate or short cylindric (Q = 2.2–5.2), 38–75 × 13.5–30 μm; paraphyses cylindric-obtuse, 3–4 μm wide or markedly swollen apically, 3–10 μm wide; apothecia entirely sessile and broadly attached to the substrate	**9**
8	Asci 125–150 × 12.5–17.5 μm, 8-spored; ascospores narrowly ellipsoid (Q = 1.7–1.9), 14–16 × 7.5–10 μm; paraphyses uncinate to helicoid	***C. uncinatus* Yei Z. Wang**
–	Asci 85–130 × 9–13 μm, 4-spored; ascospores broadly ellipsoid (Q = 1.1–1.3), 8.7–10.1 × 6.9–7.8 μm; paraphyses ±straight	***C. tetrasporus* Häffner, nom. inval.**
9	Asci short cylindric (Q = 3.8–5.2), 60–75 × 13.5–15.5 μm; living mature ascospores bilaterally symmetric, subphaseoliform to phaseoliform, 7.9–9.6 × 4.8–5.6 μm; paraphyses of two types: (a) epithecioid, apically short-celled, capitate, 6.8–10 × 5–9.9 μm, often also bi- to tri-moniliform celled, forming protective layer over immature asci, and (b) narrowly clavate 2.3–4.4 μm wide	***C. epithecioides* Matočec & I. Kušan**
–	Asci broad clavate (Q = 2.2–3.4), 38–60 × 14–30 μm; living mature ascospores ±radially symmetric, ellipsoid or oblong, 9–14.4 × 5–9.5 μm; paraphyses of a single type, apically cylindric obtuse to clavate and long-celled, 3–8 μm wide, not forming protective layer over immature asci	**10**
10	Ascospores ellipsoid to narrowly-ellipsoid (Q = 1.4–1.8), 9.5–14.5 × 6–9.5 μm; paraphyses apically bent, clavate, 4–8 μm wide	***C. granuliformis* (P. Crouan & H. Crouan) Kimbr.**
–	Ascospores narrowly oblong (Q = 1.7–2), 9–14 × 5–6 μm; paraphyses cylindric-obtuse and ±straight, apically 3–4 μm wide	***C. trichosuri* A.E. Bell & Kimbr.**
11	Number of spores in each ascus is a ±multiple of 8 in powers of two (i.e. 16, 32, 64 or ~256)	**12**
–	Asci 8-spored	**17**
12	Asci 16-spored	**13**
–	Asci with 32, 64 or ~256 spores	**14**
13	Asci clavate, 90–140 × 20–30 μm; ascospores 11–16 × 7–10 μm	***C. sexdecimsporus* (P. Crouan & H. Crouan) Kimbr. & Korf**
–	Asci cylindric, 70–90× 10–18 μm; ascospores 7.5–10 × 4–6.5 μm	***C. duplus* Kimbr., Luck-Allen & Cain**
14	Asci 32-spored	**15**
–	Asci 64 or ~256 spores	**16**
15	Asci broad clavate (Q ca. 3.5), 100–175 × 48–75 μm; ascospores narrowly ellipsoid (Q = 1.6–1.8), 13.5–17 × 7–8 μm; paraphyses filiform, apically bent and branched, up to 2 μm wide	***C. rhyparobioides* (Heimerl) Kimbr.**
–	Asci clavate (Q = 4.8–6.0), 75–112 × 19–30 μm; ascospores elongated ellipsoid (Q = 1.9–2.2) 10–12.5 × 5–7.5 μm; paraphyses apically clavate and unbranched, 5–6 μm wide	***C. albidus* (Boud.) Kimbr.**
16	Asci 64-spored, ^*^140–165 × 30–60, ^†^80–130 × 28–40 μm; paraphyses filiform, usually simple, 2–2.5 μm wide	***C. niveus* (Fuckel) Kimbr., Luck-Allen & Cain**
–	Asci ~256-spored, 160–210 × 45–55 μm; paraphyses filiform, apically branched, 1–2 μm wide	***C. winteri* (Marchal & É.J. Marchal) Kimbr.**
17	Apothecial margin beset with partially protruding prismatic terminal cells exceeding 15 μm and reaching 25 μm in length	**18**
–	Apothecial margin smooth, composed of elongated cells up to 15 μm in length, not protruding from the surface	**19**
18	Apothecia greyish-brown; ascospores broadly ellipsoid (Q = 1.2–1.4) with obtuse ends, 12–16 × 9–11.5 μm; paraphyses filiform, 2–2.5 μm wide	***C. sarangpurensis* K.S. Thind & S.C. Kaushal**
–	Apothecia white to yellowish; ascospores ellipsoid to narrowly ellipsoid (Q = 1.4–1.9) with tapered ends, 10–14 × 5–9 μm; paraphyses apically clavate, 3–4 μm wide	***C. disculus* Kimbr., Luck-Allen & Cain**
19	Paraphyses always contain abundant globular to granular yellow or orange to reddish pigment; apothecia always vividly yellow, orange or reddish-orange	**20**
–	Paraphyses lacking yellow, orange or reddish pigment, may contain refractive but hyaline globules or cytoplasm completely non-refractive and hyaline; apothecia hyaline, whitish to creamy-greyish, often becoming yellowish	**29**
20	Ascospores ±bilaterally symmetric, loaf-shaped (Q = 1.7–2.3), 14–17.3 × 6.5–8.9 μm; paraphyses markedly swollen apically, 3–8 μm wide	***C. subcylindrosporus* J. Moravec**
–	Ascospores ±radially symmetric, ellipsoid, narrowly ellipsoid or oblong; paraphyses filiform, apically not inflated to cylindric-clavate, not exceeding 5 μm in width	**21**
21	Apothecia often reaching 1 mm in diam. or more; ectal excipulum of large celled *textura globulosa-angularis* with basal cells 20–45 μm diam.; asci 100–190 μm in length	***C. ochraceus* (P. Crouan & H. Crouan) J. Moravec**
–	Apothecia seldom exceeding 0.5 mm diam. (at most 0.8); ectal excipulum composed of smaller cells, 5–24 μm diam.; asci 45–120 μm long	**22**
22	Ascospores oblong (Q = 1.5–1.8), with broadly rounded ends, very large, 17–25 × 11–14 μm	***C. vicinus* (Boud.) Kimbr., Luck-Allen & Cain**
–	Ascospores not exceeding 18.5 μm in length and 11.5 μm in diam, either broadly oblong (Q = 1.4–1.6) or ellipsoid to narrowly ellipsoid	**23**
23	Ascospores 11.5–18.5 μm long; paraphyses apically straight to bent and markedly swollen, 3–5.5 μm wide	**24**
–	Ascospores 8–12 μm long; paraphyses apically uncinate and filiform, 1.5–3.5 μm wide	**27**
24	Asci cylindric (Q = 6.1–9.5), 75–140 × 12–17 μm; ascospores 12–15 × 6–9 μm; paraphyses frequently branched above	***C. aurora* (P. Crouan & H. Crouan) K.S. Thind & Waraitch**
–	Asci short cylindric or broad clavate to clavate (Q = 2.5–4.7), 45–95 × 17–30 μm; ascospores exceeding 9 μm in width; paraphyses simple or branched near the base	**25**
25	Asci clavate (Q ~4–4.7), 80–90 × 17–20 μm; ascospores broadly oblong (Q = 1.4–1.6), 11.5–16 × 8.5–10 μm	“***Ascophanus***” ***aurantiacus* Velen.**
–	Asci broad clavate or short cylindric (Q = 2.5–3.9), 20–30 μm wide; ascospores ellipsoid to narrowly ellipsoid (Q = 1.4–1.8), always exceeding 16 μm in length	**26**
26	Asci often with only 6–7 fully matured spores, broad clavate, 60–115 × 22–30 μm; ascospores with obtuse ends, 16–18.5 × 10–11.5 μm	***C. bilobus* (Velen.) J. Moravec**
–	Asci regularly 8-spored, short cylindric, 45–60 × 20–28 μm; ascospores with tapered ends, 12.5–18 × 7.5–12 μm	***C. breviascus* (Velen.) Kimbr., Luck-Allen & Cain**
27	Asci broad clavate (Q = 3.8–4.1), 45–65 × 11–15 μm	***C. breviascus* ss. Dokmetzian et al. (Dokmetzian et al. 2005)**
–	Asci cylindric (Q = 6.2–10.0), 60–105 × 10–17 μm	**28**
28	Ascospores with obtuse ends, 8–11 × 4.5–7 μm; paraphyses apically 2–3.5 μm wide	***C. luteus* Kimbr.**
–	Ascospores with tapered ends, 10.5–12 × 6.5–7.5 μm; paraphyses apically 1.5–2 μm wide	***C. aff. luteus* (cf. Doveri 2004)**
29	Asci longer than 90 μm *or* ascospores exceed 13.5 μm in length and always broader than 7.5 μm; paraphyses apically notably swollen, clavate	**30**
–	Asci shorter than 90 μm; ascospores shorter than 13.5 μm and narrower than 7 μm; paraphyses filiform or cylindric-obtuse, apically not inflated	**32**
30	Asci broad clavate (Q = 2–3.8), 55–90 ×14.5–24 μm; ascospores ±bilaterally symmetric, hemiellipsoid i.e. with regular ellipsoid outline in dorsoventral view and inequilateral ±loaf-shaped outline in lateral view, 10.5–16 × 8.5–10.5 μm; paraphyses ±straight, not containing refractive content; apothecia turbinate, minute, up to 0.2 mm diam.; ectal excipulum composed of small globose to angular cells up to 10 μm diam.	***C. argenteus* (Curr.) Waraitch**
–	Asci clavate or short cylindric to cylindric-ventricose (Q = 3.9–6), 80–125 μm long; ascospores ±radially symmetric, ellipsoid to narrowly ellipsoid; paraphyses predominantly apically bent, usually with hyaline to subhyaline refractive content; apothecia discoid to lenticular, always exceeding 0.2 mm diam. at maturity; ectal excipulum contains globose to angular cells 4–17 μm diam., cyanophilic and dextrinoid	**31**
31	Asci clavate; ascospores 11–13.2 × 7.3–10 μm	***C. dextrinoideus* Kimbr., Luck-Allen & Cain**
–	Asci short cylindric to cylindric-ventricose; ascospores 14–18 × 7.5–11.5 μm	***C. leucopocillum* Kimbr., Luck-Allen & Cain**
32	Asci broad clavate (Q = 2.2–2.3), 50–60 × 20–26 μm; ascospores narrowly oblong (Q = 1.7–2), 9–14 × 5–6 μm; paraphyses cylindric-obtuse and ±straight; apothecia minute, 125–175 μm diam., known from dung of *Trichosurus vulpecula*	***C. trichosuri* A.E. Bell & Kimbr.**
–	Asci clavate, short cylindric to cylindric-ventricose (Q = 4–8), 7–20 μm diam.; ascospores broadly to narrowly ellipsoid or loaf-shaped (bilaterally symmetric) (Q = 1.1–1.8), 6–10 × 5–7 μm; paraphyses filiform and straight to uncinate; apothecia 0.2–1 mm diam., known from dung of placental mammals, ruminants and rodents	**33**
33	Ascospores broadly ellipsoid (Q = 1.1–1.3), 8–8.5 × 5.5–6 μm; paraphyses ±straight; ectal excipulum composed of small globose to angular cells up to 6.5 μm diam.	***C. sphaerosporus* J.L. Gibson & Kimbr.**
–	Ascospores ellipsoid to narrowly ellipsoid or loaf-shaped (Q = 1.4–1.8); paraphyses always uncinate; ectal excipulum contains cyanophilic globose to angular cells 4–15 μm diam.	**34**
34	Asci clavate (Q = 4.0–4.8), 40–70 × 7–14 μm; ascospores ±bilaterally symmetric, hemiellipsoid (i.e. ellipsoid to significantly more flattened on one side) with obtuse ends, 6–10 × 3.5–5.8 μm; paraphyses above 2.9–4.3 μm wide; apothecial margin with elongated cells up to 10 μm long	***C. glaucellus* (Rehm) Kimbr.**
–	Asci short cylindric to cylindric-ventricose (Q = 4–8), 65–95 × 12–20 μm; ascospores radially symmetric, ellipsoid to narrowly ellipsoid with tapered ends, 7.5–13 × 5–7 μm; paraphyses above 1.5–3 μm wide; apothecial margin with elongated cells 8–17.5 μm long	***C. lacteus* (Cooke & W. Phillips) Kimbr., Luck-Allen & Cain**

**Table 2.** *Coprotus* species overview - macroscopy and ecology.

Species	Apothecial shape	Apothecial diam. / mm	Pigmentation variation	Substrate / dung of:
*C. albidus* (**1**, 29)	glob-lent	0.2–0.7	always hyaline to creamy-grey	*Bos, Lepus, Felis, Canis*
*C. arduennensis* (**2**)	cup-disc	0.5–1.5	light orange	*Sus scrofa*
*C. argenteus* (**3**, 4)	obpyr-disc	~0.1–0.2	always hyaline to white	ruminants
*C. aurora* (1, **5**, 6, 7, 8, 9, 28, 29)	glob-disc	0.2–0.7	always yellow-orange	ruminants, rodents
“*Ascophanus*” *aurantiacus* (**10**, 11)	lent	0.3–0.6	always orange	*Bos*
*C. baeosporus* (**12**)	cup-disc	0.2–0.65	white to yellowish	*Cervus*
*C. bilobus* (**10**, 11, 13)	turb-lent	0.1–0.6	always yellow, orange to rosy	*Bos*
*C. breviascus* (1, **10**, 11)	disc-lent	0.2–0.6	always yellow to orange	ruminants
*C. breviascus* ss. Dokmetzian et al. (14)	disc-lent	0.2–0.6	always yellowish-orange	*Equus*
*C. dextrinoideus* (**1**, 15, 29)	cup-disc	0.1–0.5	whitish, becoming yellowish	ruminants, *Lepus*
*C. dhofarensis* (**16**)	glob-cup	0.3–0.7	orange to brownish-orange	*Capra*
*C. disculus* (**1**, 8, 9, 17, 18, 29)	disc-lent	0.3–1	hyaline to white, becoming yellowish	ruminants, rodents, *Sus*
*C. duplus* (**1**)	cup-disc	0.3–0.8	white to yellowish	ruminants, rodents, birds
*C. epithecioides* (this paper)	lent	0.2–0.4	white to yellow	*Rupicapra rupicapra*
*C. glaucellus* (1, **7**, 8, 13, 29)	disc-lent	0.2–1	white to yellow	ruminants, rodents
*C. granuliformis* (1, 7, 8, 18, **19**, 29)	glob-lent	0.2–0.6	whiite to yellowish	ruminants
*C. lacteus* (1, 7, 8, 9, 14, 17, 18, **20**, 21, 22, 29)	glob-lent	0.2–0.6	white to yellowish-ochre	ruminants, rodents
*C. leucopocillum* (**1**, 8, 9, 18, 29)	cup-lent	0.2–0.5	white to yellowish	ruminants, rodents
*C. luteus* (**1**, 9, 18, 29)	disc-lent	0.2–0.8	always yellow to orange	rumninants
*C. aff. luteus* (8)	disc-lent	0.2-0.3	yellowish	ruminants
*C. marginatus* (**1**)	disc-lent	1–1.6	white to yellowish	ruminants, rodents
*C. niveus* (**1**, 9, 14)	cup-disc	0.2–0.5	white to yellowish	various dung types
*C. ochraceus* (1, **5**, 6, 8, 9, 14, 26)	glob-disc	0.5–1.8	always yellow to orange or ochraceous	ruminants
*C. ochraceus* ss. Thind et al. (7, 17, 18)	disc-lent	0.5–1	yellow	mix of dung & *Quercus*/*Cedrus* foliage
*C. rhyparobioides* (1, 14)	glob-disc	0.1–0.4	always hyaline to white	ruminants, *Lepus*
*C. sarangpurensis* (**17**)	disc	≤0.5	always greyish-brown	*Bos*
*C. sexdecimsporus* (1, 6, 8, 14, 18, **19**, 26, 27, this paper)	disc-lent	0.5–0.8	white to yellowish	ruminants, rodents, *Sus*
*C. sphaerosporus* (**23**)	glob-disc	0.2–0.7	always hyaline to white	*Equus*
*C. subcylindrosporus* (8, 10, **13**)	disc-lent	0.3–1	always yellow to orange or rosy	ruminants, *Lepus*
*C. tetrasporus* (**27**)	disc-substip	0.2–0.4	whitish to rosy	*Lepus* (or ?*Capra*)
*C. trichosuri* (**24**)	n/a	0.1–0.2	always hyaline to white	*Trichosurus vulpecula*
*C. uncinatus* (**25**)	disc-substip	0.5–0.7	white to yellowish	*Bos*
*C. vicinus* (1, **6**)	glob-lent	0.3–1	always ochraceous to greyish-rosy	*Bos*
*C. winteri* (1)	glob-cup	0.4–0.5	always hyaline to white	ruminants

# Literature sources: 1 - Kimbrough et al. (1972), 2 - De Sloover (2002), 3 - Currey (1864), 4 - Waraitch (1977), 5 - Crouan and Crouan (1867), 6 - Boudier (1869), 7 - Rehm (1896), 8 - Doveri (2004), 9 - Melo et al. (2015), 10 - Velenovský (1934), 11 - Svrček (1976), 12 - Jeng and Krug (1977), 13 - Moravec (1971), 14 - Dokmetzian et al. (2005), 15 - Doveri (2012), 16 - Gene et al. (1993), 17 - Thind et al. (1978), 18 - Aas (1983), 19 - Crouan and Crouan (1858), 20 - Cooke (1877), 21 - Kish (1974), 22 - Chang and Wang (2009), 23 - Gibson and Kimbrough (1980), 24 - Bell and Kimbrough (1973), 25 - Wang (1994), 26 - Le Gal (1960), 27 - Häffner (1996), 28 - Thind and Waraitch (1970), 29 - data obtained from own material collected in various localities across Croatia and Slovenia during 1998–2011, deposited in CNF, bold-face - original description (same for Tables 2–6); glob - globose, lent - lenticular, cup - cupulate, disc - discoid, obpyr - obpyriform, turb - turbinate, subst - substipitate, turb - turbinate.

**Table 3.** *Coprotus* species overview - apothecial structure.

**Species**	**Medullary excipulum**	**Ectoexcipular cell diam. / µm**	**Marginal structure**	**Marginal cell dim. / µm**
*C. albidus* (**1**, 29)	red txt intr	5–12	elongated cells	2.4–4.3 diam.
*C. arduennensis* (**2**)	(–)	10–45	globose + paraphysiform	< 200
*C. argenteus* (**3**, 4)	(–)	≤ 10	elongated cells	n/a
*C. aurora* (1, **5**, 6, 7, 8, 9, 28, 29)	red txt intr	7–24	elongated cells	8–12×5–6
“*Ascophanus*” *aurantiacus* (**10**, 11)	(–)	≤ 16	elongated cells	n/a
*C. baeosporus* (**12**)	dev txt intr	3–9+cort	elongated cells	n/a
*C. bilobus* (**10**, 11, 13)	(–)	6–20	elongated cells	12–18×5–11
*C. breviascus* (1, **10**, 11)	(–)	≤ 15	elongated cells	n/a
*C. breviascus* ss. Dokmetzian et al. (14)	(–)	n/a	elongated cells	n/a
*C. dextrinoideus* (**1**, 15, 29)	(–)	3–16.8	elongated cells	8–15×3–7
*C. dhofarensis* (**16**)	dev glob-ang	15–26	raised, paraphysiform	60–95×6.5–14
*C. disculus* (**1**, 8, 9, 17, 18, 29)	(–)	5–20	elongated cells	10–24×2.5–10
*C. duplus* (**1**)	(–)	10–12	elongated cells	10–12×4–6
*C. epithecioides* (this paper)	red txt intr	5–12	±isodiametric cells	3.8–6 diam.
*C. glaucellus* (1, **7**, 8, 13, 29)	red txt intr	4–14	elongated cells	< 10 long
*C. granuliformis* (1, 7, 8, 18, **19**, 29)	(–)	5.5–22	±isodiametric cells	5.3–13.2 diam.
*C. lacteus* (1, 7, 8, 9, 14, 17, 18, **20**, 21, 22, 29)	(–)	4–15	elongated cells	8–17.5×4–10
*C. leucopocillum* (**1**, 8, 9, 18, 29)	(–)	4–17	elongated cells	12–15×3–8.4
*C. luteus* (**1**, 9, 18, 29)	(–)	10–20	elongated cells	8–12×4–5
*C. aff. luteus* (8)	(–)	5–10	elongated cells	n/a
*C. marginatus* (**1**)	(–)	12–15	raised, paraphysiform	> 100 long
*C. niveus* (**1**, 9, 14)	(–)	5–7	elongated cells	12–15×6–7
*C. ochraceus* (1, **5**, 6, 8, 9, 14, 26)	(–)	25–52	elongated cells	12–14×6–8
*C. ochraceus* ss. Thind et al. (7, 17, 18)	dev txt intr	≤ 56×45	±isodiametric cells	n/a
*C. rhyparobioides* (1, 14)	(–)	n/a	elongated cells	8–10×3–4
*C. sarangpurensis* (**17**)	dev txt intr-epi	≤ 25×20	elongated cells	< 25×8
*C. sexdecimsporus* (1, 6, 8, 14, 18, **19**, 26, 27, this paper)	red	7–12	elongated cells	5–13.2×2.5–6
*C. sphaerosporus* (**23**)	(–)	5–6.5	elongated cells	6–8.5×2–3.5
*C. subcylindrosporus* (8, 10, **13**)	(–)	8–30	elongated cells	n/a
*C. tetrasporus* (**27**)	(–)	7–14	±isodiametric cells	n/a
*C. trichosuri* (**24**)	(–)	n/a	n/a	n/a
*C. uncinatus* (**25**)	(–)	5–20	±isodiametric cells	n/a
*C. vicinus* (1, **6**)	(–)	≤ 14	elongated cells	8–11×6–8
*C. winteri* (1)	(–)	n/a	elongated cells	10–12×4–5

# (-) almost lacking / not clearly differentiated from ectal eaxcipulum, red - reduced, txt intr - *textura intricata*, dev - well developed, glob-ang - *textura globulosa-angularis*, txt intr-epi - *textura intricata-epidermoidea*.

**Table 4.** *Coprotus* species overview - ascus characters.

Species	Shape	Q	Dimensions / µm	Number of spores
*C. albidus* (**1**, 29)	clavate	4.8–6	75–112×19–30	32
*C. arduennensis* (**2**)	narrow cylindric	~10–11	150–185×10–16	8(16)
*C. argenteus* (**3**, 4)	broad clavate	2–3.8	55–90×14.5–24	8
*C. aurora* (1, **5**, 6, 7, 8, 9, 28, 29)	cylindric	6.1–9.5	75–140×12–17	8
“*Ascophanus*” *aurantiacus* (**10**, 11)	clavate	~4–4.7	80–90×17–20	8
*C. baeosporus* (**12**)	narrow cylindric	~10–11.5	69–90×6–9	8
*C. bilobus* (**10**, 11, 13)	broad clavate	2.9–3.2	60–115×22–30	6–7(8)
*C. breviascus* (1, **10**, 11)	short cylindric	2.5–3.9	45–60×20–28	8
*C. breviascus* ss. Dokmetzian et al. (14)	broad clavate	3.8–4.1^§^	45–65×11–15^§^	8
*C. dextrinoideus* (**1**, 15, 29)	clavate	4.3–6	80–125×16–24	8
*C. dhofarensis* (**16**)	cylindric	8.4–9.8	70–98×10–13	8
*C. disculus* (**1**, 8, 9, 17, 18, 29)	short cylindric to cylindric-ventricose	4–8	60–120×10–16	(4)8
*C. duplus* (**1**)	cylindric	?	70–90×10–18	16
*C. epithecioides* (this paper)	short cylindric	3.8–5.2	60–75×13.5–15.5	8
*C. glaucellus* (1, **7**, 8, 13, 29)	clavate	4–4.8	40–70×7–14	8
*C. granuliformis* (1, 7, 8, 18, **19**, 29)	broad clavate	2.3–2.9	38–58×14–20	8
*C. lacteus* (1, 7, 8, 9, 14, 17, 18, **20**, 21, 22, 29)	short cylindric to cylindric-ventricose	4–8	65–95×12–20	8
*C. leucopocillum* (**1**, 8, 9, 18, 29)	short cylindric to cylindric-ventricose	3.9–5.1	80–120×14–24	8
*C. luteus* (**1**, 9, 18, 29)	cylindric	7.5–10	55–95×10–15	8
*C. aff. luteus* (8)	cylindric	6.2–7.6	75–105×10–15	8
*C. marginatus* (**1**)	cylindric-ventricose	~9–9.5	80–100×8–12	8
*C. niveus* (**1**, 9, 14)	broad clavate	2–3	(+)80–130×28–40	64
*C. ochraceus* (1, **5**, 6, 8, 9, 14, 26)	cylindric	4–6.9	100–190×16–28	8
*C. ochraceus* ss. Thind et al. (7, 17, 18)	narrow cylindric	~14	163–200×10–16	8
*C. rhyparobioides* (1, 14)	broad clavate	~3.5–3.6	100–175×48–75	32
*C. sarangpurensis* (**17**)	cylindric	~6.6–6.7	89–115×12–16	8
*C. sexdecimsporus* (1, 6, 8, 14, 18, **19**, 26, 27, this paper)	clavate	4.1–5.6	90–140×20–30	16
*C. sphaerosporus* (**23**)	cylindric	~4.5–6	76–89×13–20	8
*C. subcylindrosporus* (8, 10, **13**)	cylindric-ventricose	5.6–6.3	80–120×15–25	8
*C. tetrasporus* (**27**)	cylindric	8.2–9.7	85-130×9-13	4
*C. trichosuri* (**24**)	broad clavate	2.2–2.3	50–60×20–26	8
*C. uncinatus* (**25**)	cylindric	~8.2–8.6	125–150×12.5–17.5	8
*C. vicinus* (1, **6**)	broad clavate	3.1–4	65–100×20–28	8
*C. winteri* (1)	clavate	n/a	160–210×45–55	256

# clavate series - maximal width in upper ¼: broad clavate - Q = 2.00–4.00, clavate - Q = 4.01–6.00, cylindric-subclavate - Q = 6.00–10.00; cylindric series - width ±uniform in upper ⅘: narrow-cylindric - Q >10.00, cylindric - Q = 5.01–10.00, short cylindric - Q = 3.00–5.00; fusiform series - maximal width in central ⅓: oblong-fusiform - Q = 3.00–4.00, cylindric-ventricose - Q > 4.00. ^§^Data derived exclusively from microphotographs.

**Table 5.** *Coprotus* species overview - ascospore characters.

Species	Symmetry	Shape	Poles	Dimensions / µm	Q
*C. albidus* (**1**, 29)	radial	elongated-ellipsoid	tapered	10–12.5×5–7.5	1.9–2.2
*C. arduennensis* (**2**)	radial	ellipsoid	tapered	12.5–15.5×6.5–7.5	1.5–1.9
*C. argenteus* (**3**, 4)	bilateral	hemiellipsoid	obtuse	10.5–16×8.5–10.5	1.4–1.8
*C. aurora* (1, **5**, 6, 7, 8, 9, 28, 29)	radial	ellipsoid - narrowly-ellipsoid	subobtuse	12–15×6–9	1.4–1.6
“*Ascophanus*” *aurantiacus* (**10**, 11)	radial	broadly-oblong	obtuse	11.5–16×8.5–10	1.4–1.6
*C. baeosporus* (**12**)	radial	elongated-ellipsoid	subobtuse	7–8.5×3.5–4.5	1.9–2.2
*C. bilobus* (**10**, 11, 13)	radial	ellipsoid - narrowly-ellipsoid	obtuse	16–18.5×10–11.5	1.4–1.8
*C. breviascus* (1, **10**, 11)	radial	ellipsoid - narrowly-ellipsoid	tapered	12.5–18×7.5–12	1.4–1.8
*C. breviascus* ss. Dokmetzian et al. (14)	radial	narrowly-ellipsoid	tapered	9.8–11.1×6.5–7.2	1.7–1.8
*C. dextrinoideus* (**1**, 15, 29)	radial	ellipsoid	subobtuse	11–13.2×7.3–10	1.4–1.8
*C. dhofarensis* (**16**)	radial	narrowly-ellipsoid	tapered	10–13.5×6–7	1.8–2
*C. disculus* (**1**, 8, 9, 17, 18, 29)	radial	ellipsoid - narrowly-ellipsoid	tapered	10–14×5–9	1.4–1.9
*C. duplus* (**1**)	radial	ellipsoid	tapered	7.5–10×4–6.5	1.5–1.8
*C. epithecioides* (this paper)	bilateral	subphaseoliform - phaseoliform	obtuse	7.9–9.6×4.8–5.6	1.5–1.9
*C. glaucellus* (1, **7**, 8, 13, 29)	bilateral	hemiellipsoid	obtuse	6–10×3.5–5.8	1.4–1.8
*C. granuliformis* (1, 7, 8, 18, **19**, 29)	radial	ellipsoid - narrowly-ellipsoid	obtuse	9.5–14.5×6–9.5	1.4–1.8
*C. lacteus* (1, 7, 8, 9, 14, 17, 18, **20**, 21, 22, 29)	radial	ellipsoid - narrowly-ellipsoid	tapered	7.5–13×5–7	1.4–1.8
*C. leucopocillum* (**1**, 8, 9, 18, 29)	radial	ellipsoid - narrowly-ellipsoid	obtuse	14–18×7.5–11.5	1.4–1.8
*C. luteus* (**1**, 9, 18, 29)	radial	ellipsoid - narrowly-ellipsoid	obtuse	8–11×4.5–7	1.4–1.9
*C. aff. luteus* (8)	radial	ellipsoid - narrowly-ellipsoid	tapered	10.5–12×6.5–7	1.5–1.8
*C. marginatus* (**1**)	radial	elongated-ellipsoid	obtuse	8.5–10×4–5	2–2.2
*C. niveus* (**1**, 9, 14)	radial	narrowly-ellipsoid	tapered	8–12×4–7.5	1.5–1.9
*C. ochraceus* (1, **5**, 6, 8, 9, 14, 26)	radial	ellipsoid - narrowly-ellipsoid	tapered	14–18.5×9–12	1.4–1.8
*C. ochraceus* ss. Thind et al. (7, 17, 18)	radial	narrowly-ellipsoid	obtuse	13.7–18×7.5–9	1.8–2
*C. rhyparobioides* (1, 14)	radial	narrowly-ellipsoid	obtuse	13.5–17×7–8	1.6–1.8
*C. sarangpurensis* (**17**)	radial	broadly-ellipsoid	obtuse	12–16×9–11.5	1.2–1.4
*C. sexdecimsporus* (1, 6, 8, 14, 18, **19**, 26, 27, this paper)	radial to slightly bilateral	ellipsoid - narrowly-ellipsoid	obtuse	11–16×7–10	1.3–1.8
*C. sphaerosporus* (**23**)	radial	broadly-ellipsoid	obtuse	8–8.5×5.5–6	1.1–1.3
*C. subcylindrosporus* (8, 10, **13**)	bilateral	loaf-shaped	obtuse	14–17.3×6.5–8.9	1.7–2.3
*C. tetrasporus* (**27**)	radial	broadly-ellipsoid	obtuse	8.7–10.1×6.9–7.8	1.1–1.3
*C. trichosuri* (**24**)	radial	narrowly-oblong	obtuse	9–14×5–6	1.7–2
*C. uncinatus* (**25**)	radial	narrowly-ellipsoid	tapered	14–16×7.5–10	1.7–1.9
*C. vicinus* (1, **6**)	radial	oblong	obtuse	17–25×11–14	1.5–1.8
*C. winteri* (1)	radial	narrowly-ellipsoid	obtuse	10–11×5–6	n/a

# Radially symmetric spores - after Kušan et al. (2014); bilaterally symmetric homopolar spores: hemiellipsoid with one side significantly to nearly flattened - Q = 1.4–1.8, loaf-shaped with one side significantly to nearly flattened - Q = 1.81–2.30; subphaseoliform with one side entirely flattened to slightly concave - Q = 1.31–1.70, phaseoliform with one side entirely flattened to slightly concave - Q = 1.71–2.00.

**Table 6.** *Coprotus* species overview - paraphysis characters.

Species	Apices	Width / µm	Branching	Bending	Refractive globules	Pigments
*C. albidus* (**1**, 29)	clavate	5–6	below	uncinate	none	none
*C. arduennensis* (**2**)	cylindric	6–9	below	straight	orange	orange globs
*C. argenteus* (**3**, 4)	cylindric-clavate	≤ 4.5	simple	straight	none	none
*C. aurora* (1, **5**, 6, 7, 8, 9, 28, 29)	cylindric-clavate	3–5	mostly above	bent	yellow, orange to reddish	globs or granules
“*Ascophanus*” *aurantiacus* (**10**, 11)	cylindric-clavate	3–5	below	bent	orange	n/a
*C. baeosporus* (**12**)	filiform	n/a	branched	bent	yellowish	yellowish
*C. bilobus* (**10**, 11, 13)	cylindric-clavate	2.5–5.5	branched	straight - bent	orange	granules
*C. breviascus* (1, **10**, 11)	cylindric-clavate	3–4	simple	straight - bent	yellowish	n/a
*C. breviascus* ss. Dokmetzian et al. (14)	filiform	1.5–2	n/a	uncinate	yellowish	granules
*C. dextrinoideus* (**1**, 15, 29)	cylindric-clavate	1.5–4.3	branched	straight to bent	hyaline - subhyaline	none
*C. dhofarensis* (**16**)	filiform	2–3	simple	straight	hyaline	none
*C. disculus* (**1**, 8, 9, 17, 18, 29)	cylindric-clavate	3–4	below	straight to bent	none	none
*C. duplus* (**1**)	filiform	2.2–2.5	below	uncinate	hyaline	none
*C. epithecioides* (this paper)	epithecioid+ cylindric-clavate	5–9.9^*^	branched	bent	±	carotenoid
*C. glaucellus* (1, **7**, 8, 13, 29)	filiform	2.9–4.3	branched	uncinate	none to yellow	none to yellow
*C. granuliformis* (1, 7, 8, 18, **19**, 29)	clavate	4–8	below	bent	none to diffuse	none to yellow
*C. lacteus* (1, 7, 8, 9, 14, 17, 18, **20**, 21, 22)	filiform	1.5–3	below	uncinate	hyaline to yellow	globs
*C. leucopocillum* (**1**, 8, 9, 18, 29)	cylindric-clavate	2–5	below	bent	none or hyaline	none
*C. luteus* (**1**, 9, 18, 29)	filiform	2–3.5	below	bent	yellow to orange	globs
*C. aff. luteus* (8)	filiform	1.5–2	mostly above	uncinate	yellow	yellow globs
*C. marginatus* (**1**)	filiform	2–3	below	bent	none	none
*C. niveus* (**1**, 9, 14)	filiform	2–2.5	below	straight to bent	none	none
*C. ochraceus* (1, **5**, 6, 8, 9, 14, 26)	cylindric-clavate	3–5	below	straight to bent	yellow	granules
*C. ochraceus* ss. Thind et al. (7, 17, 18)	cylindric-clavate	3.5–5	simple	straight	yellow	yellow content
*C. rhyparobioides* (1, 14)	filiform	1.8–2	mostly above	bent	none	none
*C. sarangpurensis* (**17**)	filiform	2–2.5	below	straight	n/a	n/a
*C. sexdecimsporus* (1, 6, 8, 14, 18, **19**, 26, 27, this paper)	filiform	1.7–3.5	branched	bent to uncinate	hyaline or pigmented	none
*C. sphaerosporus* (**23**)	filiform	n/a	below	straight	hyaline	none
*C. subcylindrosporus* (8, 10, **13**)	clavate	3–8	below	straight to bent	yellow	yellow content
*C. tetrasporus* (**27**)	filiform	1.5-2	branched	straight	hyaline	n/a
*C. trichosuri* (**24**)	cylindric-obtuse	3–4	branched	straight	none	none
*C. uncinatus* (**25**)	filiform	2–3	branched	uncinate - helicoid	n/a	n/a
*C. vicinus* (1, **6**)	cylindric-clavate	4–5	below	straight	yellow	yellow globs
*C. winteri* (1)	filiform	1–2	mostly above	uncinate	none	none

## Discussion

Together with the newly described species, 29 species are currently accepted in the genus *Coprotus*. One species is published invalidly (Häffner 1996), while four misapplied species concepts were recognized in our study and considered as separate taxonomic entities: *Ascophanus aurantiacus* Velen. (Velenovský 1934, Svrček 1976), which is erroneously synonymised by Kimbrough et al. (1972) with *Coprotus aurora* (P. Crouan & H. Crouan) K.S. Thind & Waraitch (Thind and Waraitch 1970); *Coprotus breviascus* (Velen.) Kimbr., Luck-Allen & Cain ss. Dokmetzian et al. (2005); C. aff. luteus Kimbr. (Doveri 2004) and *C. ochraceus* (P. Crouan & H. Crouan) J. Moravec ss. Thind et al. (1978). Furthermore, Kimbrough et al. (1972) synonymised *Ascophanus bilobus* Velen. (≡ *Coprotus bilobus* (Velen) J. Moravec) with *Coprotus ochraceus*, an entity we consider a separate species.

In this, our first contribution to the knowledge of the genus *Coprotus*, we aimed to ascertain the exact phylogenetic position of the genus, bearing in mind that the type species *C. sexdecimsporus* had not previously been sequenced. We also undertook to determine the variability in colour noted in this species. To do this a typical non-pigmented sample of *C. sexdecimsporus* and a pigmented 16-spored *Coprotus* collection were analysed using molecular and vital taxonomic methods. The non-pigmented *C. sexdecimsporus* and the pigmented form proved to be the same species with 100% bp identity, showing that the apothecia of *C. sexdecimsporus* may be pigmented or not. The same behaviour regarding pigmentation was also recorded in the newly described *C. epithecioides* by performing the same light-test procedure through prolonged monitoring of apothecia on original substrate. The apothecia of both *C. sexdecimsporus* and *C. epithecioides*, fully grown in dark first, were devoid of any notable pigmentation in the paraphyses, while new generations of apothecia started to develop pigment granules soon after exposure to sunlight or artificial light rich in UV radiation. This would indicate that future testing along these lines on other species in the genus would be fruitful and informative in further developing the identification key. All *Coprotus* keys published so far, that containing significant numbers of species (Kimbrough et al. 1972, Aas 1983, Prokhorov 1998, Doveri 2004, Melo et al. 2015) use paraphysal and apothecial pigmentation that we show are unstable/unreliable.

Phylogenetic analyses of both forms of the type species confirmed the position of the genus *Coprotus* in the order Pezizales, inside a large species group of the Pyronemataceae s.l., placing the *Coprotus-Boubovia* lineage next to the *Ascodesmis* species group but without high support in our contracted analyses (cf. also Hansen et al. 2013, Lindemann et al. 2015, Lindemann and Alvarado 2017). In our study *C. epithecioides* clustered in the *Coprotus* core group (sister to the type species). Our analysis confirmed that both eight-spored and multispored (in our case 16-spored) species belong in the genus *Coprotus* (cf. Hansen et al. 2013).

Previously only *C. ochraceus* was included in phylogenetic analyses (cf. Hansen et al. 2013, Lindemann et al. 2015, Lindemann and Alvarado 2017). In our analyses, this species clearly falls outside both the *Coprotus* core group and the group containing putative members of the genus *Boubovia* (Figs 1, 2). The isolated position of *C. ochraceus* is furthermore supported by the detailed re-examination of Crouan’s material by Le Gal (1960), who managed to observe several to many granules inside the sporoplasm that could not represent de Bary bubbles, a feature that is absent in all other known *Coprotus* species. However, paraphyletic relationship of analysed members of *Boubovia* should be clarified in future studies with more species and more DNA regions included. A number of *Coprotus* species (but not *C. ochraceus*) that we have studied so far in detail, including the type species *C. sexdecimsporus* and the new species *C. epithecioides*, did not possess any refractive granular / guttulate content in the sporoplasm at any developmental stage (see also Kimbrough 1966, Kimbrough and Korf 1967). All known species of *Coprotus* are obligatory fimicolous (cf. Doveri 2011). Those species in the closely related genus *Boubovia*, that were included in our phylogenetic analyses, placed next to each other (Figs 1, 2), are principally found on other types of substrate (dump soil, pebbles, litter and decayed organic material), and their ascospores possess internal guttules, at least during the early stages of development (Svrček 1977, Yao and Spooner 1996). The present study implies the necessity for further phylogenetic studies of more *Coprotus* collections and species (reliably identified), as well as more DNA regions. Until more research is done, we restrict the genus to strictly fimicolous species, the spores of which are smooth under the light microscope, and are devoid of any internal refractive granular content at any developmental stage. Also, freshly ejected ascospores of all the species analysed by us possessed thick and sticky temporary sheaths in the living state, a rarely reported, but important character, also detected by Le Gal (1960). An example of the importance of such a character in generic characterisation is the encapsulating, rather firm spore sheath present in the genus *Paratricharina* Van Vooren, U. Lindemann, M. Vega, Ribes, Illescas & Matočec (VanVooren et al. 2015) but absent from almost all pezizalean genera.

Since the need for the standardisation of defining taxonomic characters (especially spore shapes) is already elaborated in Kušan et al. (2014), we tested the shape of the asci as a useful taxonomic character too. The asci of the genus *Coprotus* vary considerably in both shape (from broad clavate to narrow cylindric) and size (38–210 × 6–55 μm) (Table 4). However, individual species in this genus mostly possess asci with comparatively little variation in size and shape. This prompted us to introduce a standardisation of ascus shape types and length/width ratio (“Q” value) for describing asci, in order to enhance differentiation between *Coprotus* species. Ascus shape types were grouped in the current study into three series, defined by the position of its broadest point and “Q” value: clavate, cylindric and fusiform (see explanation under the Table 4).

Baral (1992) observed that considerable alterations in quantitative taxonomic characters between dead and living cells exist in Ascomycota, due to the turgor loss causing cell shrinkage (especially in hymenial elements). This phenomenon, resulting in significantly lower measurements in dead cells, was recorded during the current study in ascal length and width (frequently with altered length/width ratio), and paraphysal width in all *Coprotus* collections studied in the living state. Therefore, great care should be taken when measuring the asci and paraphyses in order not to mix up the measurements of living and dead cells. On the other hand, ascospores in *Coprotus* showed little quantitative alteration. This can be explained by rigid spore walls and the capability of the sporoplasm to reversibly reduce its volume (caused by loss of cytoplasmic water) by forming gaseous de Bary bubble without significant cell shrinkage. This behaviour is not only characteristic to the genus *Coprotus*, but also to other phylogenetically closely related genera such as *Boubovia* (cf. Kristiansen and Schumacher 1993) and *Lasiobolus* Sacc. (cf. Kimbrough and Korf 1967). The ascospores of a number of more distantly related fungi usually possess pliant and thin walls, that easily irreversibly collapse unilaterally, together with the sporoplasm (e.g. *Peziza*, *Iodophanus* or *Morchella*), or both the wall and the sporoplasm irreversibly shrink, decreasing the ascospore’s size ±evenly in all parts (numerous species of *Helotiales*), as shown diagrammatically in Baral (1992).

We recommend that future studies of newly collected material of *Coprotus* include careful observations of microscopic characters in the living state, especially in cases of rare and potentially new species, for the following reasons: (1) Living mature asci, besides representing a valuable standard for measurement and shape definition, may with proper orientation display useful characteristics related to the dehiscence apparatus as it appears immediately before spore ejection. This is also the case if living material is directly fixed with CB (Fig. 5a) or CR; (2) Freshly ejected ascospores are normally at a uniform ontogenetic, mature stage, structurally complete and presumably viable, thus in this condition they represent a valuable standard for measurement, vital staining and description of structural features. Spores shape is unaltered because they are fully hydrated. This allows the differentiation of bilateral symmetry from those spores that may appear to have bilateral symmetry because of collapse due to the loss of turgor. We repeatedly recorded this alteration not only in this genus but throughout different pezizalean taxa; (3) A spontaneous (natural) spore discharge from living mature asci enables the monitoring of the presence and properties of the ascospore sheath. This structural detail can be of great help in taxonomical studies of every single species putatively assigned to the genus *Coprotus*, as well as to related taxa. It is already known that the presence or absence of such structures represents important taxonomic information in a number of ascomycetous taxa; (4) Both the paraphysal internal pigmentation and the exudate may disappear in older dried material. Observation of shrunken paraphysis tips on dead material minimises the difference among a number of species. All the above-mentioned characters, are only visible in the living state. However, they can be easily recorded (e.g. microphotography) for future use from every fresh and viable collection.

## Supplementary Material

XML Treatment for Coprotus

XML Treatment for Coprotus sexdecimsporus

XML Treatment for Coprotus epithecioides
